# Poverty, Dietary Intake, Intestinal Parasites, and Nutritional Status among School-Age Children in the Rural Philippines

**DOI:** 10.3390/tropicalmed2040049

**Published:** 2017-09-21

**Authors:** Allen G. Ross, Keren Papier, Ruby Luceres-Catubig, Thao N. Chau, Marianette T. Inobaya, Shu-Kay Ng

**Affiliations:** 1 Menzies Health Institute Queensland, Griffith University, Gold Coast, QLD 4222, Australia; net_inobaya@yahoo.com (M.T.I.); s.ng@griffith.edu.au (S.-K.N.); 2 National Centre for Epidemiology & Population Health, Australian National University, Canberra, ACT 2601, Australia; keren.papier@anu.edu.au; 3 Department of Health, Research Institute for Tropical Medicine, Muntinlupa 1781, Metro Manila, Philippines; ruby.luceres@yahoo.com; 4 Discipline of Public Health, School of Health Sciences, Flinders University, Adelaide, SA 5001, Australia; tnpchau@gmail.com

**Keywords:** childhood, malnutrition, intestinal parasites, nutritional status, poverty

## Abstract

Intestinal helminths are endemic throughout the Philippines; however, there is limited evidence with respect to their prevalence, intensity, and impact on children’s nutritional status. A cross-sectional survey was carried out on 693 children from five rural villages in Northern Samar, the Philippines. Data on dietary intake, nutritional status, and intestinal parasites were collected. Infection with *Schistosoma japonicum*, *Ascaris lumbricoides*, *Trichuris trichiura*, and hookworm was evident in 20.1, 54.4, 71.4, and 25.3% of the children. The majority (84.7%) was infected with one or more helminth species, with about one-quarter of the sample (24.7%) infected with three or more. About half (49.2%, *n* = 341) of the children were stunted and 27.8% (*n* = 193) were wasted. A lower prevalence of normal height-for-age (48.3%) appeared in those with polyparasitism, while the prevalence of stunted children increased with infection (46.7% monoparasitism and 51.7% polyparasitism). There was a decreasing trend between infection intensity and the mean values of HAZ and BAZ identified for *T. trichiura* or hookworm infections. Stunted children were more likely to be male (AOR = 1.58; 95% CI: 1.05–2.39; *p* = 0.028), older in age (10–14 years) (AOR = 1.93; 95% CI: 1.29–2.88; *p* = 0.001), and living in poorer households with palm leaves/nipa roof (AOR = 1.85; 95% CI: 1.14–3.01; *p* = 0.013). Intestinal parasitic treatment needs to be combined with nutrient supplements and health education in order to interrupt the parasite life cycle and achieve sustainable control.

## 1. Introduction

More than a third of the world’s population is infected with soil-transmitted helminths (STH), mainly in the developing nations of Asia, Africa, and Latin America [1]. STHs are intestinal parasitic nematode worms causing human disease. They are the most common of the 17 major neglected tropical diseases (NTDs) and the most widespread and disabling chronic infections globally [2]. *Ascaris lumbricoides* is the most prevalent STH with an estimated one billion infections; and *Trichuris trichiura* and hookworms (*Necator americanus* and *Ancylostoma duodenale*) each infect approximately 600–800 million [1]. STHs are a significant public health concern in the Philippines, particularly among school-aged children who, if infected, suffer from profound physical deficits, including anaemia and malnutrition, stunted growth, reduced fitness, and cognitive delays [2,3,4,5,6,7,8]. Sixteen out of 17 regions in the Philippines are endemic for STHs with a prevalence of ≥50% [9]. A nationwide survey performed over 10 years found the prevalence in children aged 2–14 years was 50–90%; and up to 30% of the 22 million children in the Philippines were infected with more than one of the three STH species [10,11].

The cornerstone of intestinal parasitic control is recurrent mass drug administration (MDA) with benzimidazole anthelmintics (e.g., 400 mg albendazole) that are cheap, safe, and effective. The current WHO strategy is to continually treat pre-school and school-age children, women of childbearing age, and adults at high risk, once or twice per year, depending on prevalence [8,9]. This is partially effective in achieving morbidity control; however, it does not prevent re-infection. A number of studies have shown that once treatment is stopped, prevalence returns to pre-treatment levels within 12–18 months [2,7,8,9,10,11]. Therefore, interventions that prevent re-infection and boost immunity (e.g., the use of micro/macronutrient supplements) are required to augment chemotherapy as part of an integrated approach. The global target is to eliminate morbidity due to soil-transmitted helminthiases in children by 2020 [8]. This will only be achieved by regularly treating at least 75% of the children in endemic areas (an estimated 873 million), who are free from malnutrition [8,9].

Nutritional deficiencies and infectious diseases can negatively impact the nutritional status of children and adolescents [12,13]. Intestinal helminth worm infections can damage a child’s internal mucosa, leading to impaired digestion and poor absorption of nutrients [14]. Deficiencies in macro- and micronutrient intakes during childhood can impair both physical and cognitive growth as well as increase the risk of mortality [15]. Moreover, inadequate intake of selected micronutrients can cause immune deficiency and increase susceptibility to infection [16]. The micronutrients vitamin A, vitamin B_12_, vitamin C, β-carotene, riboflavin, zinc, selenium, and iron all have immune-modulating functions, enabling them to influence the course of an infection [16]. Laboratory studies have shown that vitamin A deficiency can reduce schistosome (human blood fluke)-specific antibody responses, suggesting a possible link between vitamin A deficiency and susceptibility to schistosomiasis [12]. Deficiency of some nutrients may reduce the host’s immune function, impairing the body’s resistance to infectious diseases and increasing susceptibility to intestinal parasites [17]. Once present, parasitic infections can promote the further loss of nutrients, leading to reduced growth and poor nutritional status as part of a vicious cycle [18]. Children aged 5–14 years suffer from the highest burden of infectious disease [19], partly due to their increased behavioural risk, frequent outdoor exposure, and poor personal hygiene [20].

Intestinal helminths are endemic throughout the Philippines and efforts are underway to decrease their burden. However, there is limited evidence with regards to their prevalence, intensity and their impact on children’s nutritional status. The purpose of this study was to examine the relationship between poverty, dietary intake, intestinal parasites, and childhood nutritional status in the rural Philippines.

## 2. Material and Methods

### 2.1. Study Population

A cross-sectional survey was conducted in 2013 on 693 children from five rural villages in Palapag [21], Northern Samar, the Philippines. Villagers there are typically poor rice farmers, with over 50% of the population living below the poverty line. Water, sanitation, and hygiene conditions are most often rudimentary. Most households typically have 6–10 children per family and the prevalence rates of parasitic diseases, acute respiratory infections, diarrhoeal diseases, and other communicable diseases, are high [22].

### 2.2. Study Procedures

Individuals were asked, over the course of a week, to provide two stool specimens from which six Kato–Katz thick smears were prepared on microscope slides. These slides were examined under a light microscope by experienced laboratory technicians who counted the number of STH and *Schistosoma japonicum* (SJ) eggs per slide. For quality control, 10% of slides were randomly selected and re-examined by a senior microscopist at the Research Institute for Tropical Medicine, Manila. Individual and head of household questionnaires were completed to collect the following information: occupation, level of education, home and land ownership, number of animals owned and raising practices, animal waste disposal practices, pasturing of animals, sanitation, and housing characteristics (roofing, wall, and floor materials). For wealth status, participants were classified as wealthy if their house had a cement floor, a galvanized roof, cement walls, and a tile/marble floor. Participants were classified as poor if they had a house with a nipa (palm) roof and a soil floor, and without cement walls. All other participants were classified as having a moderate wealth status.

### 2.3. Nutritional Assessment

Anthropometric measurements of height and weight were collected using standard procedures [23]. Weight was measured using a portable digital scale to the nearest 0.1 kg. Height was assessed to the nearest 0.1 cm using a tape measure. The Z values for weight-for-height (WAZ) (children aged <10 years only), body mass index (BMI)—for-age (BAZ), and height-for-age (HAZ) were calculated according to World Health Organization (WHO) guidelines using the new WHO growth standards [24,25]. Weight-for-height is considered an inappropriate indicator for monitoring child growth beyond the age of 10 due to its inability to distinguish between relative height and body mass. Therefore, BMI-for-age was used to assess thinness/wasting for children aged ≥10 and for adolescents. Based on the Z values, the children were categorized as ‘thin/wasted’ (BAZ < −2 and/or WAZ < −2) and ‘stunted’ (HAZ < −2). Children with Z values > −2 for BAZ, WAZ, and HAZ were categorized as ‘normal’.

### 2.4. Dietary Intake Data

Dietary intake information was elicited using a 24-h recall method. Three qualified nutritionists together with 10 field nurses collected the data. Household food utensils were used to assist study participants quantify food portions and liquids consumed. In order to estimate food weights, macro- and micronutrient intakes were calculated for each child using food composition tables developed by the Food and Nutrition Research Institute [26]. These tables contained data on 17 food components of 1541 foods commonly consumed in the Philippines. Dietary intake data was evaluated against the national Filipino recommended energy and nutrient intake (RENI) values by age and sex [27].

### 2.5. Statistical Analysis

Data were double-entered into FoxPro (version 6.0), crosschecked, and subsequently analysed using STATA SE version 13.0 software (StataCorp LP, College Station, TX, USA). All variables including sex, age group, and endemic setting were explored individually by Chi-square statistics. Infection intensity was explored with the Student *t*-test and Kruskal–Wallis test. The standard error (SE) of each estimate was converted to a variance; all variances were summed to provide an overall variance, SE, and 95% confidence interval (CI). The Chi-square test and the Student *t*-test were used to explore associations of a participant’s demographic and socio-economic characteristics and the likelihood of having *S. japonicum*, any STH, and any helminth infection. Significant demographic and socio-economic factors were entered into the mixed-effect logistic regression analysis to obtain the final model for predicting stunting. Random barangay (village) and household effects were included in the model to account for the correlation among observations within each barangay and household, respectively. Adaptive Gaussian quadrature with 10 points was adopted to approximate the log likelihood for all levels of both random effects in the mixed model. Factors that were not significantly relevant (cut-off for significance = 0.05) were removed in a stepwise backward regression elimination procedure.

### 2.6. Study Oversight

Ethical consent for the study was obtained from the ethics review boards of the Department of Health in the Philippines (IRB # 2012-13-0) and Griffith University, Australia. Written informed consent was obtained from the parents/legal guardians. All questionnaires were translated into the local dialect and back-translated into English. Individuals found positive for a STH or *S. japonicum* were treated according to the Department of Health clinical guidelines.

## 3. Results

### 3.1. Demographic, Household, and Nutritional Characteristics and Prevalence of Infection

A cross-sectional survey was carried out on 693 children, of whom 53% were male. A total of 41.7% of the study population was aged between 6–9 years with the remainder between 10–14 years. The majority of children (56%) lived in a house with a roof made from either palm leaves or nipa, an indirect indicator of lower socioeconomic status. Infection with *S. japonicum*, *Ascaris lumbricoides*, *Trichuris trichiura*, and hookworm was evident in 20.1, 54.4, 71.4, and 25.3% of the 667 children sampled for intestinal parasites. The majority of the children (84.7%) was infected with one or more helminth species, with about one-quarter of the study sample (24.7%) infected with three or more different worm species.

The demographic, household, and nutritional characteristics of the study sample are presented in Table 1. About half (49.2%, *n* = 341) of the study sample were stunted and 27.8% (*n* = 193) were thin. Both mean HAZ and BAZ scores were below world standard (−2.0 SD and −1.3 SD from world mean, respectively). SJ infection occurred more often for males (64.2%, *p* = 0.003) and higher age group (70.2%, *p* = 0.002). Children with *S. japonicum* infection also had lower BAZ scores (−1.603, *p* = 0.039). Children with any STH infection were more likely to be of higher age group (60.5%, *p* = 0.019) or living in houses with palm leaves/nipa roofs (57.5%, *p* = 0.040). There was no significant difference for the nutrition indicators between children with and without any STH infection. Age group and roof material were the only factors that differentiated the three children groups of non-infected, monoparasitism, and polyparasitism (proportion of polyparasitism was higher for the higher age group, *p* = 0.018, and for those with house roof materials of palm leaves or nipa, *p* = 0.021).

### 3.2. Demographic, Household, and Nutritional Characteristics and Intensity of Infection

Table 2 presents the demographic, household, and nutritional characteristics of the study sample, by intensity of infection. Significant results were found between negative, light, and moderate/heavy SJ infection. Males (*p* = 0.01) and older children (*p* = 0.009) were more likely to have *S. japonicum* infection. For *A. lumbricoides* infections, children in the household without toilets (*p* = 0.009) or without galvanized iron/cement roof (*p* <0.001) were more likely to have moderate or heavy infections. These factors have the same impact for *T. trichiura* infections. Children in households without toilets (*p* < 0.001) or galvanized iron/cement roof (*p* = 0.038) were more likely to have moderate or heavy infections. Moreover, children with moderate or heavy *T. trichiura* infections had a significantly higher mean levels of vitamin C intake, compared to those with light infections (*p* = 0.038). Finally, children with light hookworm infections were more likely to be male (62.1% versus 49.4%, *p* = 0.004), to be stunted (61.5% versus 44.8%, *p* < 0.001), and had a higher proportion of households without toilets (25.7% versus 17.9%, *p* = 0.028), and lower mean HAZ Z-scores (−2.14 versus −1.92, *p* = 0.014) and BAZ Z-scores (−1.61 versus −1.37, *p* = 0.013). 

**Figure 1:**
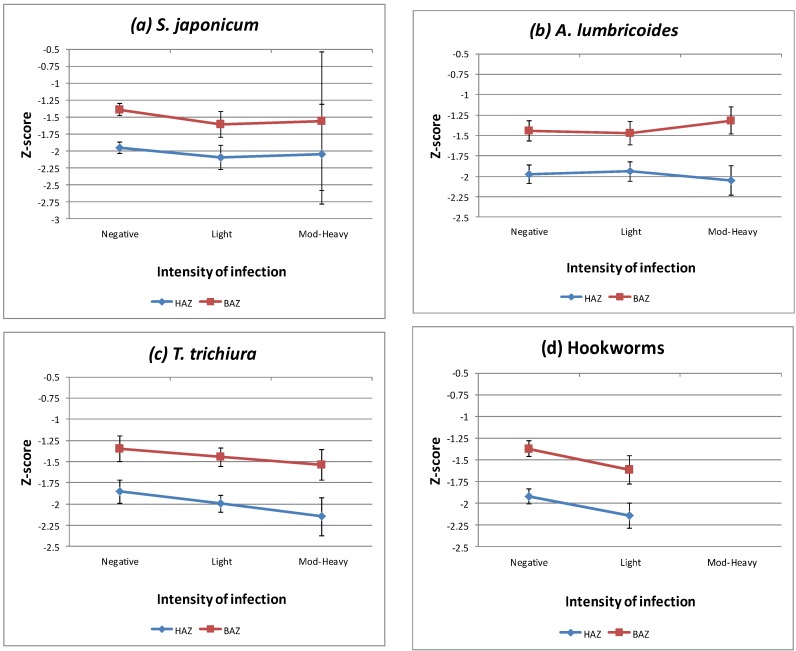
Plots of Z-scores for the anthropometric indicators versus (**a**) *Schistosoma japonicum*; (**b**) *T. trichiura*; (**c**) *A. lumbricoides*; (**d**) hookworms infection. Note HAZ: height for age Z-score; BAZ: BMI for age Z-score. Mod-Heavy: infections of moderate to heavy intensity.

As depicted in Figure 1, a decreasing trend between infection intensity and the mean values of HAZ and BAZ was identified for *T. trichiura* or hookworm infections (that is, the heavier the intensity, the lower the HAZ and BAZ mean values). For SJ or *A. lumbricoides* infections, the trend was not so obvious.

### 3.3. Demographic, Socioeconomic Factors and Stunting

Table 3 displays the mixed-effect logistic regression model for stunting. Compared to children with normal height for age, stunted children were more likely to be male (AOR = 1.58; 95% CI: 1.05–2.39; *p* = 0.028), older in the age group of 10–14 (AOR = 1.93; 95% CI: 1.29–2.88; *p* = 0.001), and living in poorer households with palm leaves/nipa roofs (AOR = 1.85; 95% CI: 1.14–3.01; *p* = 0.013). All nutrition factors were not significantly associated with stunting. Variation among the predicted barangay-specific random effects for stunting was not statistically significant. However, there is significant household-specific random effects (estimated variance: 1.82, *p* < 0.001) in the probability of stunting, indicating that unknown household effects other than the identified household risk factor (roof materials) exist.

## 4. Discussion

The current WHO strategy for intestinal helminths in children is to continually treat pre-school and school-age children at high risk once or twice per year depending on prevalence [8]. This is effective in achieving morbidity control; however, it does not prevent re-infection. Our study area has participated in national control efforts for over two decades yet the prevalence of helminth infection remains stubbornly high due largely to poverty and malnutrition. In our study, we found that approximately 85% of the rural children were infected with one or more helminth infections. *T. trichiura* infections (71.4%) were found to be more prevalent than *A. lumbricoides* (54.4%) infections. Moreover, about half (49%) of the study sample were stunted and almost a third (28%) were wasted. Stunted children were more likely to be male, older in age (10–14 years), and living in poorer households with palm leaves/nipa roofs.

In the mixed-effect logistic regression model for stunting all of the nutrition factors (i.e., grams) were found not to be significantly associated with stunting. However, we previously found a significant association between the coinfection of all four helminthiases and low intakes of energy, thiamine, and riboflavin among children, when the recommended energy and nutrient intake (RENI) for total calories was examined [21]. Thiamine and riboflavin deficiencies are common in Northern Samar, where dairy and meat intakes are low and mostly rice-based meals are consumed [21]. Iron deficiency has been associated with impairments in both adaptive and innate immunity and with lowering the body’s resistance to infectious diseases [21]. Poor nutrient intake may increase susceptibility to parasitic diseases and together they negatively affect the nutritional status of children and adolescents [21].

We believe that a deworming program must be coupled with a nutrition program at the primary school level. Children are presently eating 1–2 meals per day at home and this is insufficient to meet their macro or micronutrient requirements. An additional meal at school appears to be of paramount importance for those severely malnourished. In order to address this problem the Philippine government has initiated the school-based feeding program called ‘*Gulayan sa Paaralan*’, which has been successfully piloted in approximately one percent of schools. However, to date it has not been formally evaluated in a clinical trial.

An appropriate eight-week micronutrient weaning period of ‘ready-to-use therapeutic foods’ (RUTF), with demonstrated immune-modulating functions—including iron, zinc, calcium, vitamin A, B and C, n-3 and n-6 fatty acids—also needs to be considered following the macronutrient school intervention. In a recent pilot study conducted at the Philippine General Hospital, the researchers created their modified version of RUTF from commercially-available ingredients including milk, sugar, coconut oil, and peanut butter [28]. A total of 100 children (aged 18 months to 10 years) was randomized to either a RUTF group, who received the supplement, and a control group, who did not [28]. The treatment group received RUTF on weekdays for five weeks. Changes in weight, height, and arm circumference were recorded for five weeks and two weeks after supplementation. Results of the study showed that RUTF was an effective, safe, and acceptable alternative supplement for children with mild to severe malnutrition [28].

Annual or biannual albendazole treatment (i.e., 400 mg) needs to be combined with macro/micronutrient supplements, WASH, and health education in order to interrupt the life cycles of STH diseases, prevent reinfection, and achieve sustainable control. A well-nourished population, with an intact immune system, has a better chance of warding off future parasitic infection. Simply providing drugs to malnourished populations, which is a common practice in the global control of STHs, is not the answer. Both poverty and malnutrition must be addressed if future MDA programs for NTDs are to have a lasting impact.

## Figures and Tables

**Table 1:** Demographic, household, and nutritional characteristics of the study sample (*n* = 693) according to *Schistosoma japonicum* (SJ) and/or soil-transmitted helminth (STH) infections.

Characteristic	All Children (*n* = 693)	Positive for SJ Infection (*n* = 134)		Positive for any STH Infection (*n* = 552)		Non-Infected (*n* = 102)	Monoparasitism (*n* = 184)	Polyparasitism (*n* = 381)	*p* Value ^b^
			***p* value ^a^**		***p* value ^a^**				
Gender									
Male	365 (52.7%)	86 (64.2%)	0.003 *	294 (53.3%)	0.470	49 (48.0%)	90 (48.9%)	212 (55.6%)	0.195
Female	328 (47.3%)	48 (35.8%)		258 (46.7%)		53 (52%)	94 (51.1%)	169 (44.4%)	
Age group									
6–9	289 (41.7%)	40 (29.9%)	0.002 *	218 (39.5%)	0.019 *	55 (53.9%)	76 (41.3%)	146 (38.3%)	0.018 *
10–14	404 (58.3%)	94 (70.2%)		334 (60.5%)		47 (46.1%)	108 (58.7%)	235 (61.7%)	
Toilet									
Yes	547 (80.0%)	108 (81.2%)	0.726	434 (79.6%)	0.492	84 (83.2%)	149 (81.9%)	295 (78.5%)	0.451
No	137 (20.0%)	25 (18.8%)		111 (20.4%)		17 (16.8%)	33 (18.1%)	81 (21.5%)	
Own home									
Yes	620 (90.4%)	121 (91.0%)	0.965	497 (90.9%)	0.671	92 (91.1%)	170 (92.4%)	340 (90.4%)	0.746
No	66 (9.6%)	12 (9.0%)		50 (9.1%)		9 (8.9%)	14 (7.6%)	36 (9.6%)	
Roof material									
Palm leaves/nipa	380 (56.1%)	72 (54.1%)	0.692	312 (57.5%)	0.040 *	44 (44.9%)	97 (53.0%)	223 (59.8%)	0.021 *
Galvanized iron/cement	298 (43.9%)	61 (45.9%)		231 (42.5%)		54 (55.1%)	86 (47.0%)	150 (40.2%)	
Height for age									
Normal	352 (50.8%)	60 (44.8%)	0.108	274 (49.6%)	0.130	58 (56.9%)	98 (53.3%)	184 (48.3%)	0.235
Stunted	341 (49.2%)	74 (55.2%)		278 (50.4%)		44 (43.1%)	86 (46.7%)	197 (51.7%)	
BMI for age									
Normal	500 (72.2%)	89 (66.4%)	0.103	399 (72.4%)	0.667	71 (69.6%)	133 (72.3%)	276 (72.6%)	0.831
Thin	193 (27.8%)	45 (33.6%)		152 (27.6%)		31 (30.4%)	51 (27.7%)	104 (27.4%)	
Mean HAZ score	−1.985 (0.973)	−2.091 (0.976)	0.131	1.999 (0.986)	0.196	−1.85 (0.92)	−1.98 (1.04)	−2.01 (0.96)	0.360
Mean BAZ score	−1.285 (3.961)	−1.603 (1.099)	0.039 *	−1.448 (1.082)	0.412	−1.39 (1)	−1.34 (1.14)	−1.49 (1.05)	0.291
Mean energy (kj)	7512 (3078)	7371 (2737)	0.577	7463 (3166)	0.531	7528 (2538)	7489 (2663)	7486 (3382)	0.992
Mean protein (g)	55.3 (52.3)	52.8 (20.8)	0.304	55.48 (57.80)	0.765	54.51 (20.13)	51.35 (18.45)	57.46 (68.38)	0.435
Mean total fat (g)	36.7 (31.6)	35.6 (22.8)	0.654	36.50 (34.04)	0.911	35.95 (17.61)	37.33 (21.74)	36.18 (38.17)	0.908
Mean carbohydrate (g)	310.7 (119.7)	307.4 (124.1)	0.764	308.0 (117.2)	0.313	314.6 (121.3)	311.9 (122.7)	308.1 (116.3)	0.864
Mean water (g)	1997 (699)	2016 (661)	0.614	2016 (661.246	0.614	1989 (674)	1956 (736)	2006 (681)	0.725
Mean thiamin (g)	0.663 (1.33)	0.578 (0.292)	0.121	0.671 (1.480)	0.611	0.63 (0.33)	0.61 (0.33)	0.70 (1.77)	0.713
Mean riboflavin (mg)	0.583 (0.617)	0.532 (0.303)	0.108	0.586 (0.679)	0.769	0.59 (0.29)	0.54 (0.30)	0.60 (0.79)	0.571
Mean niacin (mg)	17.1 (10.9)	16.5 (8.5)	0.473	0.487 (11.448)	0.803	17.06 (8.77)	15.87 (7.21)	17.53 (12.84)	0.242
Mean vitamin C (mg)	36.0 (52.1)	30.4 (44.7)	0.110	36.62 (52.12)	0.645	33.94 (56.45)	39.62 (55.43)	35.13 (49.91)	0.569

Data are count (%) for categorical variables and mean (standard deviation) for continuous variables. ^a^ Test differences between participants with positive infection vs negative infection (using either *t*-test or Chi-square test). ^b^ Test differences among participants without infection, monoparasitism, and polyparasitism (2–4 infections of SJ or any STH) (using either *t*-test or Chi-square test). * Significance at the 0.05 level.

**Table 2:** Demographic, household, and nutritional characteristics of the study sample according to intensity of infection.

Characteristic	*Schistosoma japonicum* ^a^			*A. lumbricoides* ^b^			*T. trichiura* ^c^			Hookworms ^d^	
	Negative (*n* = 533)	Light (*n* = 124)	Mod-Heay (*n* = 10)	Negative (*n* = 304)	Light (*n* = 241)	Mod-Heavy (*n* = 122)	Negative (*n* = 191)	Light (*n* = 392)	Mod-Heavy (*n* = 84)	Negative (*n* = 498)	Light (*n* = 169)
Gender ^a,d^											
Male	265 (49.7%)	79 (63.7%)	7 (70.0%)	161 (53.0%)	131 (54.4%)	59 (48.4%)	99 (51.8%)	202 (51.5%)	50 (59.5%)	246 (49.4%)	105 (62.1%)
Female	268 (50.3%)	45 (36.3%)	3 (30.0%)	143 (47.0%)	110 (45.6%)	63 (51.6%)	92 (48.2%)	190 (48.5%)	34 (40.5%)	252 (50.6%)	64 (37.9%)
Age group ^a^											
6–9	237 (44.5%)	37 (29.8%)	3 (30.0%)	128 (42.1%)	98 (40.7%)	51 (41.8%)	91 (47.6%)	151 (38.5%)	35 (41.7%)	211 (42.4%)	66 (39.1%)
10–14	296 (55.5%)	87 (70.2%)	7 (70.0%)	176 (57.9%)	143 (59.3%)	71 (58.2%)	100 (52.4%)	241 (61.5%)	49 (58.3%)	287 (57.6%)	103 (60.9%)
Toilet ^b,c,d^											
Yes	420 (79.9%)	99 (80.5%)	9 (90.0%)	253 (84.1%)	190 (79.8%)	85 (70.8%)	153 (81.0%)	325 (83.6%)	50 (61.7%)	404 (82.1%)	124 (74.3%)
No	106 (20.1%)	24 (19.5%)	1 (10.0%)	48 (15.9%)	48 (20.2%)	35 (29.2%)	36 (19.0%)	64 (16.4%)	31 (38.3%)	88 (17.9%)	43 (25.7%)
Own home											
Yes	481 (91.1%)	113 (91.9%)	8 (80.0%)	278 (92.1%)	217 (90.8%)	107 (89.2%)	175 (92.1%)	353 (90.8%)	74 (90.2%)	452 (91.5%)	150 (89.8%)
No	47 (8.9%)	10 (8.1%)	2 (20.0%)	24 (7.9%)	22 (9.2%)	13 (10.8%)	15 (7.9%)	36 (9.2%)	8 (9.8%)	42 (8.5%)	17 (10.2%)
Roof material ^b,c^											
Palm leaves/nipa	292 (56.1%)	67 (54.5%)	5 (50.0%)	142 (47.5%)	137 (57.8%)	85 (72.0%)	95 (51.1%)	214 (55.2%)	55 (68.8%)	264 (54.2%)	100 (59.9%)
Galvanized iron/cement	229 (43.9%)	56 (45.5%)	5 (50.0%)	157 (52.5%)	100 (42.2%)	33 (28.0%)	91 (48.9%)	174 (44.8%)	25 (31.2%)	223 (45.8%)	67 (40.1%)
Height for age ^d^											
Normal	280 (52.5%)	56 (45.2%)	4 (40.0%)	160 (52.6%)	125 (51.9%)	55 (45.1%)	106 (55.5%)	196 (50.0%)	38 (45.2%)	275 (55.2%)	65 (38.5%)
Stunted	253 (47.5%)	68 (54.8%)	6 (60.0%)	144 (47.4%)	116 (48.1%)	67 (54.9%)	85 (44.5%)	196 (50.0%)	46 (54.8%)	223 (44.8%)	104 (61.5%)
BMI for age											
Normal	391 (73.5%)	83 (66.9%)	6 (60.0%)	212 (69.7%)	176 (73.3%)	92 (75.4%)	136 (71.2%)	284 (72.6%)	60 (71.4%)	363 (72.9%)	117 (69.6%)
Thin	141 (26.5%)	41 (33.1%)	4 (40.0%)	92 (30.3%)	64 (26.7%)	30 (24.6%)	55 (28.8%)	107 (27.4%)	24 (28.6%)	135 (27.1%)	51 (30.4%)
Mean HAZ score ^d^	−1.95 (1.0)	−2.09 (1.0)	−2.05 (1.0)	−1.98 (1.0)	−1.94 (0.9)	−2.05 (1.0)	−1.86 (1.0)	−2.00 (1.0)	−2.15 (1.0)	−1.92 (1.0)	−2.14 (0.9)
Mean BAZ score ^d^	−1.39 (1.1)	−1.61 (1.1)	−1.56 (1.4)	−1.45 (1.1)	−1.47 (1.1)	−1.32 (0.9)	−1.35 (1.1)	−1.45 (1.1)	−1.54 (0.8)	−1.37 (1.1)	−1.61 (1.1)
Mean energy (kj)	7349 (2792)	7643 (2015)	7990 (3178)	7536 (2544)	7628 (3890)	7122 (2372)	7632 (2811)	7472 (3308)	7279 (2477)	7483 (3105)	7523 (2993)
Mean protein (g)	55.97 (58.6)	52.32 (20.8)	58.29 (20.7)	52.86 (19.0)	60.29 (84.6)	51.67 (20.1)	54.14 (20.7)	55.90 (67.2)	55.34 (21.3)	54.93 (54.8)	56.48 (48.3)
Mean totalfat (g)	36.68 (33.7)	35.80 (23.0)	32.93 (21.4)	36.57 (19.5)	38.59 (46.7)	31.99 (15.6)	38.76 (22.7)	36.65 (37.4)	30.36 (16.6)	36.73 (32.4)	35.67 (29.8)
Mean carbohydrate (g)	310.9 (117)	306.1 (126)	324.3 (99.3)	315.0 (120)	308.4 (120)	301.6 (113)	314.6 (128)	307.9 (114)	310.8 (119)	309.3 (119)	312.6 (119)
Mean water (g)	1982 (704)	2005 (672)	2158 (519)	1992 (681)	1984 (711)	1994 (705)	2005 (693)	1973 (690)	2028 (728)	1976 (694)	2028 (700)
Mean thiamin (g)	0.69 (1.51)	0.58 (0.30)	0.54 (0.22)	0.60 (0.29)	0.80 (2.22)	0.57 (0.26)	0.65 (0.38)	0.69 (1.74)	0.57 (0.24)	0.67 (1.39)	0.65 (1.24)
Mean riboflavin (mg)	0.60 (0.69)	0.53 (0.31)	0.51 (0.25)	0.54 (0.27)	0.64 (0.98)	0.57 (0.29)	0.59 (0.32)	0.57 (0.78)	0.61 (0.29)	0.58 (0.64)	0.60 (0.58)
Mean niacin (mg)	17.13 (11.5)	16.18 (8.1)	20.34 (12.3)	16.33 (7.8)	17.88 (15.0)	16.93 (7.8)	16.81 (8.4)	16.79 (12.4)	18.40 (8.7)	16.76 (11.1)	17.70 (10.8)
Mean vitamin C ^c^ (mg)	37.65 (54.2)	29.76 (44.0)	38.24 (55.0)	37.55 (55.4)	35.66 (49.1)	33.83 (51.8)	36.12 (53.2)	33.37 (47.3)	49.51 (69.7)	36.01 (51.2)	36.71 (56.2)

Data are count (%) for categorical variables and mean (standard deviation) for continuous variables. ^a^ Significant difference between negative, light, and moderate/heavy SJ infection in gender (*p* = 0.01) and age group (*p* = 0.009). ^b^ Significant difference between negative, light, and moderate/heavy *A. lumbricoides* infection in the proportions of owning toilet (*p* = 0.009) and galvanized iron/cement roof material (*p* < 0.001). ^c^ Significant difference between negative, light, and moderate/heavy *T. trichiura* infection in the proportions of owning toilet (*p* < 0.001) and galvanized iron/cement roof material (*p* = 0.028), and the mean level of vitamin C (*p* = 0.038). ^d^ Significant difference between negative and light hookworms infection in gender (*p* = 0.004), the proportions of owning toilet (*p* = 0.028) and stunted children (*p* < 0.001), and the mean levels of the HAZ Z-score (*p* = 0.014) and the BAZ Z-score (*p* = 0.013).

**Table 3:** Mixed-effect logistic regression analysis of the relationship between stunting with demographic and socio-economic variables.

Variable	Height for Age		Stunting Versus Normal	
	Normal (*n* = 352)	Stunted (*n* = 341)	Adjusted OR (95% CI)	*p*-Value
Gender				
Male	172 (48.9%)	193 (56.6%)	1.58 (1.05–2.39)	0.028
Female	180 (51.1%)	148 (43.4%)	Reference	
Age group				
6–9	167 (47.4%)	122 (35.8%)	Reference	
10–14	185 (52.6%)	219 (64.2%)	1.93 (1.29–2.88)	0.001
Roof material				
Palm leaves/nipa	173 (50.0%)	207 (62.4%)	1.85 (1.14–3.01)	0.013
Galvanized iron/cement	173 (50.0%)	125 (37.6%)	Reference	
Barangay variance			0.10 (0.01–1.25)	
Household variance			1.82 (0.91–3.64)	

## References

[1] Bethony J., Brooker S., Albonico M., Geiger S.M., Loukas A., Diemert D., Hotez P.J. (2006). Soil-transmitted helminth infections: Ascariasis, trichuriasis, and hookworm. Lancet.

[2] Hotez P.J. (2009). Mass drug administration and integrated control for the world’s high-prevalence neglected tropical diseases. Clin. Pharmacol. Ther..

[3] Brooker S., Hotez P.J., Bundy D.A. (2008). Hookworm-related anaemia among pregnant women: A systematic review. PLoS Negl. Trop. Dis..

[4] Miguel E.A., Kremer M. (2004). Worms: Identifying impacts on education and health in the presence of treatment externalities. Econometrica.

[5] Sakti H., Nokes C., Hertanto W.S., Hendratno S., Hall A., Bundy D.A. (1999). Evidence for an association between hookworm infection and cognitive function in Indonesia school children. Trop. Med. Int. Health.

[6] Nokes C., Grantham-McGregor S.M., Sawyer A.W., Cooper E.S., Robinson B.A., Bundy D.A. (1992). Moderate to heavy infections of *Trichuris trichiura* affect cognitive function in Jamaican school children. Parasitology.

[7] Hotez P. (2008). Hookworm and poverty. Ann. N. Y. Acad. Sci..

[8] World Health Organization (WHO) (2005). Deworming for Health and Development.

[9] World Health Organization (WHO) (2008). Priority Communicable Diseases: Health in Asian and the Pacific.

[10] Belizario V.Y., de Leon W.U., Lumampao Y.F., Anastacio M.B., Tai C.M. (2009). Sentinel surveillance of soil-transmitted helminthiases in selected local government units in the Philippines. Asia Pac. J. Public Health.

[11] Easton A. (1999). Intestinal worms impair child health in the Philippines. BMJ.

[12] Reilly L., Nausch N., Midzi N., Mduluza T., Mutapi F. (2012). Association between micronutrients (vitamin A, D, iron) and schistosome-specific cytokine responses in Zimbabweans exposed to *Schistosoma haematobium*. J. Parasitol. Res..

[13] Zhou H., Ohtsuka R., He Y., Yuan L., Yamauchi T., Sleigh A.C. (2005). Impact of parasitic infections and dietary intake on child growth in the schistosomiasis-endemic Dongting Lake Region, China. Am. J. Trop. Med. Hyg..

[14] Hesham M.S., Edariah A.B., Norhayati M. (2004). Intestinal parasitic infections and micronutrient deficiency: A review. Med. J. Malaysia.

[15] Katona P., Katona-Apte J. (2008). The interaction between nutrition and infection. Clin. Infect. Dis..

[16] Cunningham-Rundles S., McNeeley D.F., Moon A. (2005). Mechanisms of nutrient modulation of the immune response. J. Allergy Clin. Immunol..

[17] Nga T.T., Winichagoon P., Dijkhuizen M.A., Khan N.C., Wasantwisut E., Wieringa F.T. (2011). Decreased parasite load and improved cognitive outcomes caused by deworming and consumption of multi-micronutrient fortified biscuits in rural Vietnamese schoolchildren. Am. J. Trop. Med. Hyg..

[18] Amare B., Ali J., Moges B., Yismaw G., Belyhun Y., Gebretsadik S., Woldeyohannes D., Tafess K., Abate E., Endris M. (2013). Nutritional status, intestinal parasite infection and allergy among school children in northwest Ethiopia. BMC Pediatr..

[19] Sanza M., Totanes F.I., Chua P.L., Belizario V.Y. (2013). Monitoring the impact of a mebendazole mass drug administration initiative for soil transmitted helminthiasis (STH) control in the Western Visayas region of the Philippines from 2007 through 2011. Acta Trop..

[20] Belizario V.Y., Totanes F.I., de Leon W.U., Lumampao Y.F., Ciro R.N. (2011). Soil transmitted helminth and other intestinal parasitic infections among school children in indigenous people communities in Davao del Norte, Philippines. Acta Trop..

[21] Papier K., Williams G.M., Frauk A., Olveda R.M., McManus D.P., Harn D.A., Li Y.S., Gray D.J., Chau T.N.P., Ross A.G. (2014). Chronic malnutrition and parasitic helminth interations. Clin. Infect. Dis..

[22] Ross A.G., Olveda R.M., Chy D., Olveda D.U., Li Y., Harn D.A., Gray D.J., McManus D.P., Tallo V., Chau T.N. (2015). Can mass drug administration lead to the sustainable control of schistosomiasis?. J. Infect. Dis..

[23] Gibson R.S. (2005). Principles of Nutritional Assessment.

[24] World Health Organization (WHO) (2009). WHO AnthroPlus for Personal Computers Manual: Software for Assessing Growth of the World’s Children and Adolescent.

[25] World Health Organization (WHO) (2007). WHO AnthroPlus Software: Software for Assessing Growth and Development of the World’s Children.

[26] Philippine Statistics Authority (1997).

[27] Food and Nutrition Research Institute (2002). Recommended Energy and Nutrient Intakes.

[28] Laylo-Navarr C., Limos E. (2011). A randomized controlled trial on the efficacy and safety of a modified ready to use therapeutic food among malnourished children. Acta Med. Philipp..

